# Proximal humerus derotational osteotomy for internal rotation instability after locked posterior shoulder dislocation: early experience in four patients

**DOI:** 10.1186/s13037-015-0062-9

**Published:** 2015-05-08

**Authors:** Bruce Ziran, Ali Nourbakhsh

**Affiliations:** Gwinnett Medical Center, 575 Professional Dr., Suite 360, Lawrenceville, GA 30046 USA; Atlanta Medical Center, 303 Parkway Dr. NE, Atlanta, GA 30312 USA

**Keywords:** Humerus, Derotational osteotomy, Hill-Sachs lesion

## Abstract

**Background:**

We performed a retrospective and descriptive study to determine the feasibility of proximal humerus derotational osteotomy in younger patients with significant humeral head depression, who may not be good candidates for shoulder arthroplasty.

**Methods:**

Rotational osteotomy was done on four patients with a mean age of 40 for locked posterior dislocation associated with a reverse Hill-Sachs lesion from 2000–2011. The average age was 40 +/− 11 years old and the average follow up was 22 +/− 8 months. Shoulder stability, range of motion, radiographic outcome and postoperative complications were assessed. Average follow-up was 22 months (range, 12–30 months) postoperatively.

**Results:**

The average range of motion of the shoulders at the final follow-up were as follows (Mean +/− Standard deviation): Abduction: 125 +/− 29°, Forward flexion: 135 +/− 17°, Internal rotation: 65 +/− 17°, External rotation: 62 +/− 10°. There were no wound or neurological complications and no dislocations. Patients were satisfied with their functional status and did not have any further symptoms of instability or rotator cuff dysfunction.

**Conclusions:**

Proximal humerus derotational osteotomy for acute locked posterior dislocation of the shoulder can be a viable option for younger age group, which can facilitate rehabilitation for these patients by providing immediate stability.

## Background

Shoulder stability depends on a proper balance between the head of the humerus and the glenoid cavity as well as the soft tissues and peri-articular muscle function [1]. Posterior shoulder dislocation is usually caused by seizure, trauma or an electric shock which is the result of the internal rotators (Latissimus dorsi, Pectoralis major, and Subscapularis) overpowering the external rotators (infraspinatus and teres minor) [2]. Posterior locked shoulder dislocations are rare entities with reverse Hill Sachs lesions being a common finding [3]. Different treatment options have been described on how to restore the humerus head anatomy such as transfer of the subscapularis tendon or lesser tuberosity into the anteromedial impaction fractures [4], or joint arthroplasty [4]. Poor function, instability, and persistent pain are potential complications with transfer of the subscapularis tendon or lesser tuberosity. Subscapularis tendon transfer has been reported to fail if used for humeral head defects more than 50% [5]. Patient satisfaction with shoulder arthroplasty in younger patients has been proved to be lower than the older patients [6,7]. This has been attributed to the higher expectations of the younger age group and the fact that the reconstruction should last for a longer period of time [8].

Humerus derotational osteotomy has been described before for chronic posterior shoulder dislocations in the literature [9]. We reported the results of our humerus derotational osteotomy for acute locked posterior shoulder dislocations. The purpose of this report was to determine the feasibility of this procedure in younger patients with significant humeral head depression, who may not be good candidates for shoulder arthroplasty. As a retrospective study it was exempt from IRB approval.

## Methods

**Figure 1:**
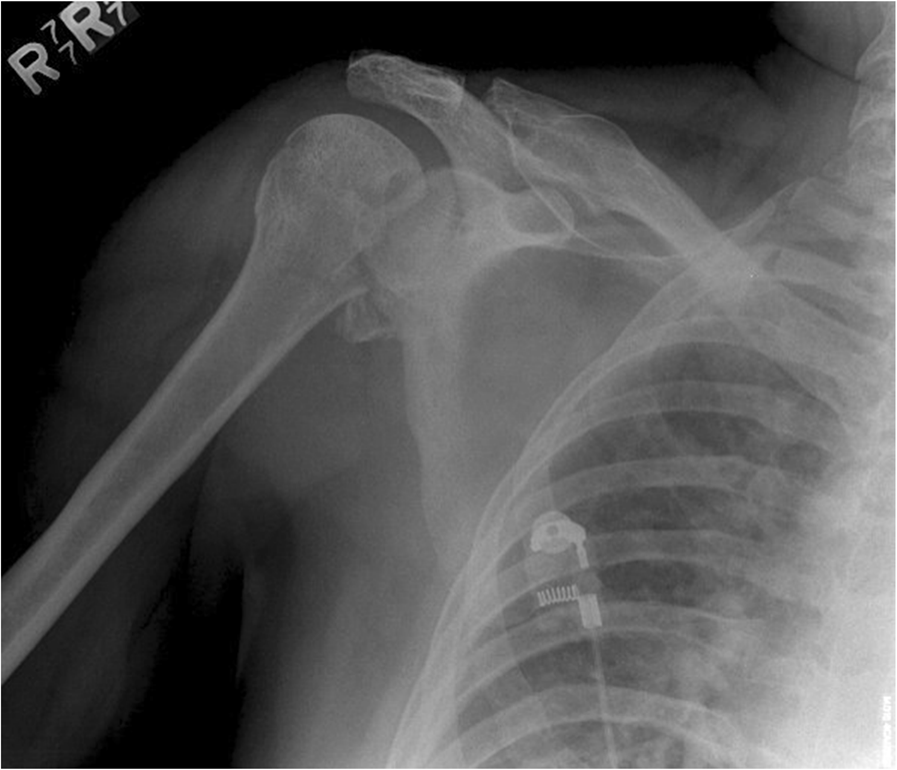
Anteroposterior view of the right shoulder with posterior dislocation and reverse Hill Sachs lesion.

**Figure 2:**
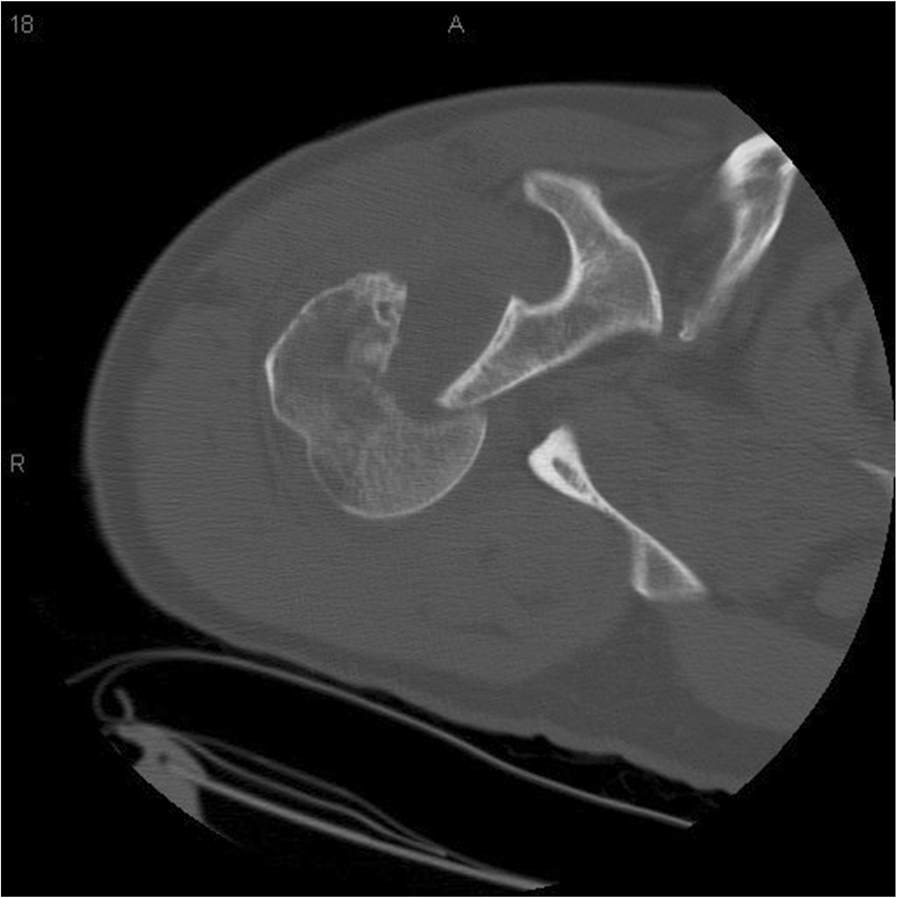
CT scan of the right shoulder axial view showing the posterior dislocation and reverse Hill Sachs lesion.

We reported the results of humerus derotational osteotomy on 4 patients from 2000 to 2011. Three patients were males and the average age was 40 +/− 11 years old (range form 31 to 56). Three dislocations were in the right and one in the left shoulder. The average duration of symptoms was 10 days. The injury mechanism was seizure in 2 patients and fall in the other two. Our average follow up was 22 +/− 8 months (12 to 30 months). We did not have any perioperative complications. Table 1 shows the demographics of these patients. The indication for performing a rotational osteotomy in all of these patients was locked posterior dislocation associated with a reverse Hill-Sachs lesion (20%-40%) with internal rotational instability (Figure 1). All patients had attempted reduction but were either irreducible (3 of 4 cases) or were unstable with any internal rotation past neutral. After appropriate radiographs, all patient had computed tomography (CT) of both shoulders (Figure 2). After the procedure, each patient had standard post-operative therapy protocol consisting of passive motion for the first six weeks, followed by active assisted motion for another six weeks, and then strengthening exercises.

**Table 1:** Summary of the demography, history and treatment of the patients

**Case**	**Age**	**Mechanism**	**Duration**	**Treatment**	**F/U**
1	35y	Fall	7d	Osteotomy 4.5 DCP non-locking	30 m
2	39y	Seizure	10d	Osteotomy 4.5 DCP non-locking + cortical graft	28 m
3	31y	Fall	17d	Osteotomy 4.5 LCP	18 m
4	56y	Seizure	7d	Osteotomy 4.5 LCP	12 m
Mean +/- STD	40 +/- 11y		10 +/- 5		22 +/- 8 m

Demographics, history and treatment of the patients. d: days, DCP: Dynamic compression plate, F/U: Follow-up, LCP: Low contact plate, m: months, STD: Standard deviation, y: years.

The patient was positioned supine on the operating table. A closed reduction was tried under anesthesia, which failed to reduce the fracture the shoulder in three patients, and in one, the shoulder remained unstable with any internal rotation past neutral. A standard deltopectoral approach was utilized for access to the proximal humerus, which was then extended distally corresponding to the anterior Henry approach. The long head of the biceps tendon was used to enter the articulation and care was taken to avoid any damage to the insertion of the subscapularis tendon. The biceps tendon was released in preparation for a tenodesis at the end of the procedure. In all the cases, the lesser tuberosity and anterior articular surface were fragmented or depressed without any chance of stable repair.

**Figure 3:**
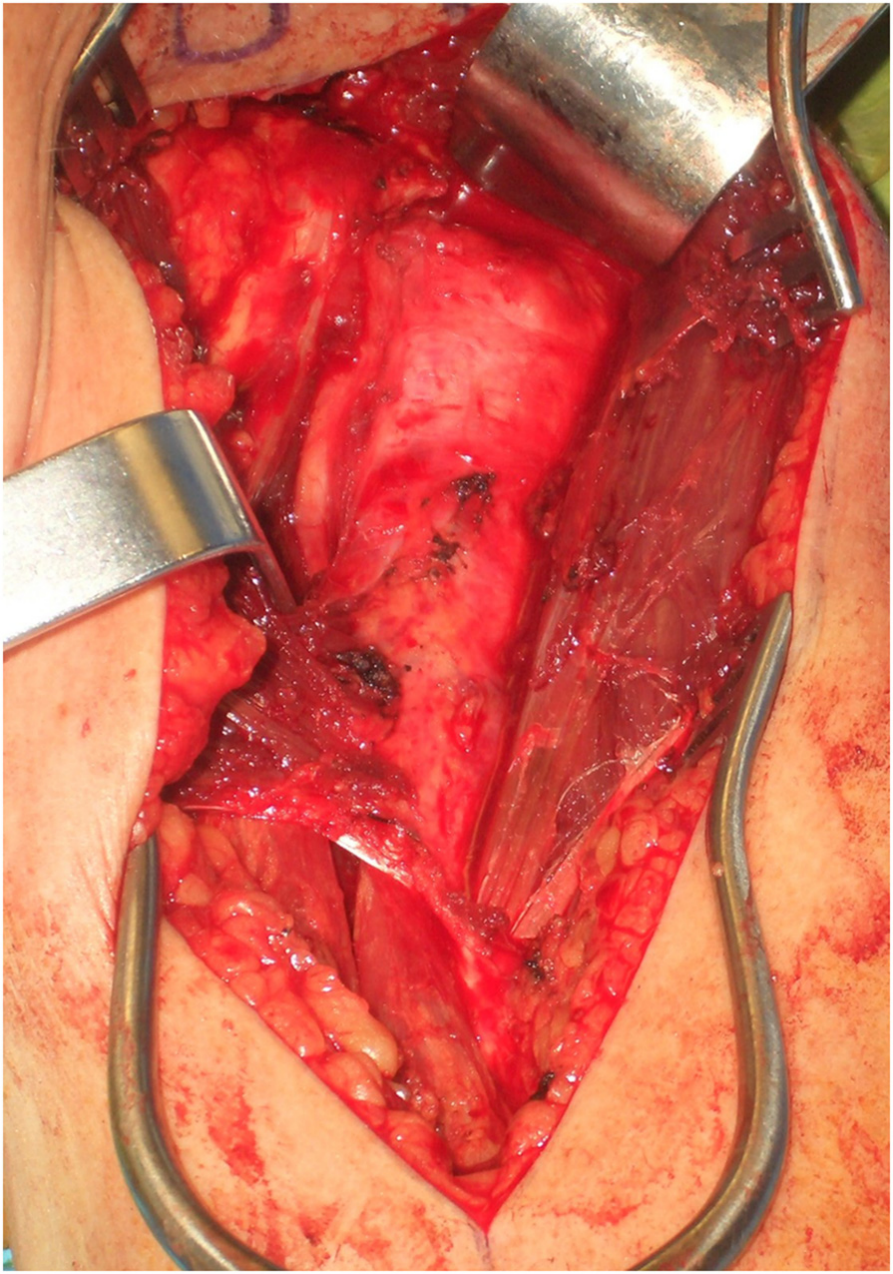
Intraoperative view of the left shoulder showing the proximal humerus prior to osteotomy.

**Figure 4:**
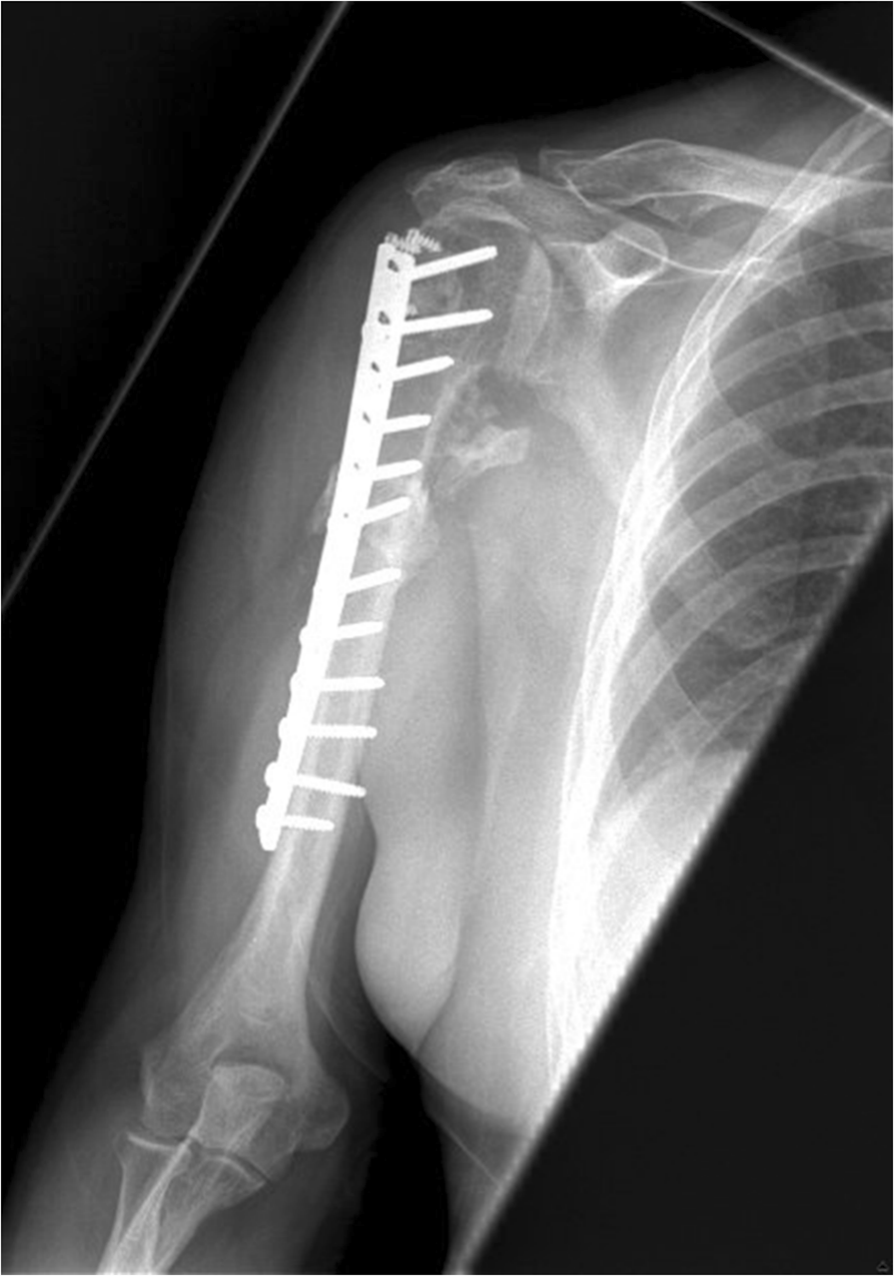
Anteroposterior view of the right shoulder after the surgery showing the well fixed implant.

Distal dissection continued to identify the insertion of the deltoid tendon, and the osteotomy site was adjacent to the insertion of the tendon. Based on CT scans, we determined that an external rotation of between 30–45 degrees was required so K wires were used in the proximal and distal segments to provide appropriate landmarks of correction. An extraperisteal transverse humerus osteotomy was performed with a water-cooled saw below the insertion of the pectoralis major and above the insertion of the deltoid tendon (Figure 3). Using the K-wire the humerus head was externally rotated and provisionally fixed with a contoured narrow 4.5 mm plate (Table 1). At that juncture, the shoulder was tested for stability and definitive fixation completed. The biceps tendon was tenodesed, and any rotator cuff issues addressed. Rotator cuff insertions were repaired with fiber wire or suture anchors. In one case, the defect was so large a cortical strut segment was placed into the defect and held under the plate (Figure 4).

## Results

Average follow-up was 22 months postoperatively. The average range of motion of the shoulders at the final follow-up were as follows (Mean +/− Standard deviation): Abduction: 125 +/− 29°, Forward flexion: 135 +/− 17°, Internal rotation: 65 +/− 17°, External rotation: 62 +/− 10°. There were no wound or neurological complications and no dislocations. Most patients had some discomfort at the terminal aspects of motion but did not complain of interference with their activities of daily living or recreational activities. Patients did not have any further symptoms of instability or rotator cuff dysfunction. At each follow up patients were asked about their satisfaction or dissatisfaction with their functional status. While we did not perform any formal functional outcome analysis, none of the patients expressed any specific dissatisfaction with their ability to perform activities of daily living and their outcome status. Manual resistance testing of muscle groups were used to asses the rotator cuff function including resisted abduction at 30 degrees, and resisted external rotation with arm at side. All of patients demonstrated clinical (no pain) and radiographic (3 out of 4 bridging cortices) by the 12 week follow up. At final follow up (22 +/− 8 months), patients were using their limb and there were no clinical or radiographic signs of failed healing and thus we concluded that all patients had healed successfully. There were no signs of existing or beginning posttraumatic osteoarthritis detectable in the standard X-rays at the final follow up.

## Discussion

Posterior locked shoulder dislocation fracture is a rare injury comprising 2–4% of all shoulder dislocations. The decision for the treatment of chronic posterior dislocation of the shoulder is based on the size of the Reverse Hill Sachs lesion [10]. The treatment can be difficult based on the fact that the head vascularization is at risk, [3] the head defect can propagate as the humerus head lies on the glenoid rim for a long time. This makes the shoulder joint more prone to future instability and osteoarthritis [3].

The treatment of a posterior dislocation is based on the size of the humeral head depression, duration of injury, the age and activity of the patient. A reverse Hill Sachs lesion of < 25% of the head can be treated by closed or open reduction in a stable shoulder or with subscapularis tendon transfer if the shoulder is unstable. A lesion between 25% and 50% of the articular surface, can be treated by lesser tuberosity transfer and if more than 50% by shoulder arthroplasty [2]. Elderly patients tolerate chronic posterior shoulder dislocation better than young patient despite the deformity and loss of rotation of the shoulder. They usually report minimal pain and enough forward flexion to allow usual functions of daily living [11].

Reconstruction of the humeral head using either a femoral head allograft [12] or an autograft of the contralateral shoulder has been described [3]. Also retrograde elevation of the depressed articular surface [13,14] and subscapularis tendon transfer for the lesions with an extent of 20–50% of the head surface [15] have been previously reported.

The shortcomings of articular cartilage elevation procedures include: (1) it might not be imperative in every case especially very chronic cases (more than 6 months),[3] (2) graft incorporation might be an issue in the face of an already compromised blood supply of the humeral head [12] and finally postoperative head collapse might ensue resulting in recurrence.

Keppler reported the use of this procedure in 10 patients with locked posterior dislocation of the shoulder in 1994. The average injury to diagnosis interval was 155 days (range, 21–400 days). The reverse Hill Sachs lesion involved 20-40% of the articular surface based on preoperative CT scan images. Postoperative complication was transient axillary nerve palsy. Six patients had good-excellent results, 2 patients had fair and 2 patients had poor results. Poor results were seen with advanced articular cartilage damage [9].

External rotation osteotomy of the humerus for posterior shoulder dislocation prevents the engagement of the large anteromedial humeral defect on the glenoid rim. Although patients will have some restriction of external rotation the average external rotation was 62° postoperatively in our case series. This might be due to gradual relaxation of the subscapularis tendon and capsule after the start of ROM exercises and better rehab potential of our younger age group. Better rehabilitation methods, better patient compliance and relatively younger age of our patient population might explain the fact that the final external rotation in our patients was better than Keppler at al. study (62° versus 7°) [9]. We did not obtain a final follow up MRI which might have indicated any adaptive changes of the rotator cuff muscles since our patients did not demonstrate any clinical indication for such a study, as well as cost/insurance concerns for coverage of such services. The osteotomy described also allows immediate postoperative rehabilitation compared to reconstructed humeral head, which has the risk of subsequent failure due to head collapse. As such, the external rotation osteotomy may be useful in younger patients who are not ideal candidates for shoulder arthroplasty [2]. Also since head collapse is not an issue with humerus derotation osteotomy, these patients may have faster rehabilitation. The use of this procedure mandates a non-arthritic articular cartilage and a reverse Hill Sachs lesion < 40% of the articular surface [9].

As compared to Keppler et al.’s study, our patients had more acute presentation with average duration of symptoms of 10 days [9]. Also the average age of patients in Keppler study was 53 years old (range, 40–78), but in our patients average age was 40 (31, 35, 39 and 56). Our patients had comparable range of motions in abduction (125° vs. 128°) and flexion (135° vs. 131°) to Keppler’s series however the internal rotation was lower (65° vs. 84°) and external rotation (62° vs. 7°) was much higher.

## Conclusions

Summarily, proximal humerus derotational osteotomy for acute locked posterior dislocation of the shoulder can be a viable option for younger age group, which can facilitate rehabilitation for these patients by providing immediate stability. As a case series, this report has the obvious limitations of: (1) the lack of a control group with other types of treatment and matched reverse Hill Sachs lesion size and age, (2) limited number of patients which is due to the rarity of posterior shoulder dislocation and high percentage of missed injuries. Further studies considering these items and shed more light on better management of these types of injuries in younger population.
